# Promoter Methylation of Selected Genes in Non-Small-Cell Lung Cancer Patients and Cell Lines

**DOI:** 10.3390/ijms21134595

**Published:** 2020-06-28

**Authors:** Victoria Sarne, Samuel Huter, Sandrina Braunmueller, Lisa Rakob, Nico Jacobi, Melitta Kitzwögerer, Christoph Wiesner, Peter Obrist, Rita Seeboeck

**Affiliations:** 1 Department Life Sciences, IMC University of Applied Sciences Krems, 3500 Krems, Austria; victoria.sarne@meduniwien.ac.at (V.S.); Sandrina.Braunmueller@fh-krems.ac.at (S.B.); Lisa.Rakob@gmx.at (L.R.); Jacobi.Nico@gmail.com (N.J.); christoph.wiesner@fh-krems.ac.at (C.W.); 2 Pathologylab Dr. Obrist & Dr. Brunhuber OG, 6511 Zams, Austria; samuel.huter@tyrolpath.at (S.H.); Peter.Obrist@tyrolpath.at (P.O.); 3 Clinical Institute of Pathology, University Hospital St. Poelten, Karl Landsteiner University of Health Sciences, 3100 St. Pölten, Austria; Melitta.Kitzwoegerer@stpoelten.lknoe.at

**Keywords:** DNA methylation, epigenetics, epigenetic marker, NSCLC, precision medicine

## Abstract

Specific gene promoter DNA methylation is becoming a powerful epigenetic biomarker in cancer diagnostics. Five genes (*CDH1*, *CDKN2Ap16*, *RASSF1A*, *TERT*, and *WT1*) were selected based on their frequently published potential as epigenetic markers. Diagnostic promoter methylation assays were generated based on bisulfite-converted DNA pyrosequencing. The methylation patterns of 144 non-small-cell lung cancer (NSCLC) and 7 healthy control formalin-fixed paraffin-embedded (FFPE) samples were analyzed to evaluate the applicability of the putative diagnostic markers. Statistically significant changes in methylation levels are shown for *TERT* and *WT1*. Furthermore, 12 NSCLC and two benign lung cell lines were characterized for promoter methylation. The in vitro tests involved a comparison of promoter methylation in 2D and 3D cultures, as well as therapeutic tests investigating the impact of *CDH1/CDKN2Ap16/RASSF1A/TERT/WT1* promoter methylation on sensitivity to tyrosine kinase inhibitor (TKI) and DNA methyl-transferase inhibitor (DNMTI) treatments. We conclude that the selected markers have potential and putative impacts as diagnostic or even predictive marker genes, although a closer examination of the resulting protein expression and pathway regulation is needed.

## 1. Introduction

Lung cancer (LC), ranked by incidence as well as mortality, is the most common type of cancer worldwide. It is categorized into two main types, small-cell LC (SCLC) and non-SCLC (NSCLC), where the latter accounts for about 85% of all new LC cases [1,2]. Recently, the incidence of LC has been decreasing in developed countries but increasing in developing regions such as Africa and South America. This divergence can be explained by the less stringent smoking regulations in developing countries [3]. However, the LC incidence also differs by sex, with LC rates among women being the highest in North America, Northern/Western Europe, and Australia/New Zealand, while the highest rates for men are found in Micronesia/Polynesia, Eastern Asia, and most of Europe [4]. In the Austrian population, the gender gap in LC trends regarding incidence and mortality is strikingly obvious. Both factors have been consistently decreasing over the past 15 years for men, whereas a steady increase was observed in the female population [5].

Due to the inherent heterogeneity of NSCLC, the treatment of each individual must be personalized according to the patient’s performance status, stage of disease, and histological and molecular profile. Therefore, molecular pathology, companion diagnostics, and the availability of reliable biomarkers are steadily gaining importance. Common oncogene modifications involved in NSCLC include the *epidermal growth factor receptor* (*EGFR*) and *KRAS Protooncogene, GTPase* (*KRAS)* genes [6]. *EGFR* mutations have been found to be more common in females [7]. Treatment with EGFR-tyrosine kinase inhibitors (TKIs) has shown a high response rate, prolonged progression-free survival, and improved quality of life compared to standard chemotherapy in eligible patients. Notably, exon 19 deletions and exon 21 L858R point mutations in the *EGFR* gene led to an increased responsiveness to TKIs. Mutations in exon 20, however, cannot be targeted by these therapies, highlighting the importance of molecular diagnostics [8]. As this pathway is central for the tumor behavior, often associated with oncogene addiction, for diagnostics and therapy design, in this work, all NSCLC analyses were performed under consideration of the EGFR/KRAS mutational background [9,10,11].

Cancer initiation and progression are based on alterations in gene expression, caused by both genetic and epigenetic modifications. Either of these can lead to the deregulation of genes involved in crucial physiological systems, ultimately driving the affected cell into tumorigenesis [12]. Due to the association of DNA promoter methylation and gene repression, hypermethylation is proposed to be an essential participant in malignant cell transformation [12,13,14,15]. Furthermore, carcinogenesis is known to have many contributing lifestyle factors, such as poor diet, physical inactivity, and obesity. The mechanisms mediating these reversible alterations are widely proposed to be epigenetic.

While the presence of physiological differences between men and women is evident, epigenomic differences between the two sexes have only recently been subjected to scientific scrutiny. Some studies have shown no difference in the autosomal DNA methylation between genders, while others have reported differentially methylated genes (DMGs), as well as differentially methylated CpG sites [16,17,18,19,20]. Nevertheless, a gender disparity in the incidence, invasiveness, and associated prognosis and mortality has been observed for various cancer entities. Predominantly, a trend towards higher overall methylation and blood global interspersed nuclear elements-1 (*LINE-1*) methylation was found in males compared to females [21,22,23,24,25,26,27]. However, the opposite has also been reported [28,29,30,31].

The five genes that are the focus of this work encode known tumor suppressors that have been suggested to be silenced by methylation in cancer: *Cyclin-Dependent Kinase Inhibitor 2A/p16* (*CDKN2Ap16*), *Telomerase Reverse Transcriptase* (*TERT)*, *RAS-association domain family 1 isoform A* (*RASSF1A*), *E-Cadherin* (*CDH1*), and *Wilms’ Tumor 1* (*WT1*).

*CDKN2Ap16* is frequently inactivated by methylation in NSCLC and has even been linked to the early-stage pathogenesis of LC [32,33,34]. CDKN2Ap16 is a tumor suppressor protein involved in the regulation of the cell cycle, senescence, apoptosis, cell invasion, and angiogenesis [35].

The *TERT* gene encodes Telomerase, which is responsible for the extension of telomeres, thereby increasing the lifespan of cells or even leading to immortalization, as commonly observed in cancer cells [36]. Different studies have found conflicting results regarding the effects of promoter hypermethylation and telomerase expression, making the role of the promoter methylation of *TERT* in carcinogenesis elusive [37].

*RASSF1A* is a tumor suppressor involved in cell proliferation and apoptosis [38]. *RASSF1A* has been found to be a major target of tumor-associated epigenetic dysregulation, as it is preferentially silenced via DNA hypermethylation rather than by mutations [39]. The promoter hypermethylation of *RASSF1A* has been closely associated with NSCLC carcinogenesis [40]. Its low physiological methylation and easy detectability in several bodily fluids make *RASSF1A* a suitable putative disease biomarker [41].

*CDH1* is a tumor suppressor gene encoding E-cadherin, which is essential for cellular adhesion and tissue morphogenesis. Hypermethylation of the E-cadherin promoter is likely the reason for cellular reprogramming that ultimately leads to epithelial–mesenchymal transition (EMT) [42,43].

Lastly, the *WT1* gene encodes a transcription factor that can act as both a tumor suppressor—inducing kidney tumors when mutated—and an oncogene that is highly expressed in many tumor types [44]. In NSCLC, the promoter methylation of *WT1* was found to be increased in neoplastic tissue. The degree of methylation was also linked to smoking status [45]. *WT1* methylation can reportedly be used as a biomarker to efficiently predict LC status [46].

In this work, we aimed to describe, in a cohort of archival NSCLC samples, the *EGFR* expression and mutation status, as well as mutations in the *KRAS* gene and the methylation status of the potential epigenetic marker genes *CDH1*, *CDKN2Ap16*, *RASSF1A*, *TERT*, and *WT1* to determine gender-specific differences. For the analysis of the latter, pyrosequencing protocols were developed and validated for applicability in molecular diagnostics. Furthermore, we scrutinized the applicability of 2D and 3D cell culture models for the investigation of DNA promoter methylation by characterizing 12 NSCLC cell lines and two benign lung cell lines. We also investigated the impact of *CDH1/CDKN2Ap16/RASSF1A/TERT/WT1* promoter methylation on sensitivity to tyrosine kinase inhibitor (TKI) and DNA methyl-transferase inhibitor (DNMTI) treatments.

## 2. Results

### 2.1. FFPE Samples vs. Cell Lines

LC is the precision oncology prototype with the highest number of reported, routinely tested, and actionable genomic alterations [47,48,49]. As the importance of epigenetic, i.e., methylation markers, is increasing, we investigated the behavior of promoter methylation in five selected marker genes in NSCLC formalin-fixed paraffin-embedded (FFPE) samples and cell lines. 

**Figure 1:**
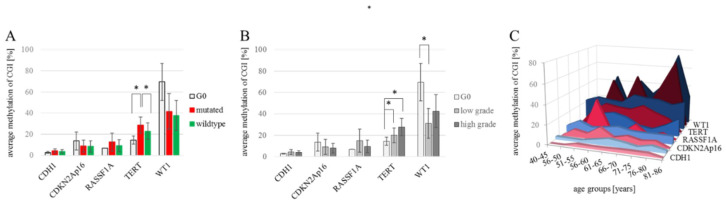
Developments of *CDH1/CDKN2Ap16/RASSF1A/TERT/WT1* methylation levels. (**A**) Average promoter methylation levels for each gene of interest for tumor-free control samples (n_CDH1_ = 2; n_CDKN2Ap16_ = 7; n_RASSF1A_ = 1; n_TERT_ = 7; n_WT1_ = 3), wildtype (n_CDH1_ = 34; n_CDKN2Ap16_ = 89; n_RASSF1A_ = 22; n_TERT_ = 78; n_WT1_ = 23) and *EGFR/KRAS* mutated tumor samples (n_CDH1_ = 22; n_CDKN2Ap16_ = 46; n_RASSF1A_ = 19; n_TERT_ = 39; n_WT1_ = 16). Significant changes are measured for *TERT* methylation; i.e., G0 vs. mutated, *p* = 0.050 and mutated vs. wild-type *p* = 0.046. Student’s t-test. (**B**) Average methylation of each gene in non-tumor/G0, low grade (G1-G2) (n_CDH1_ = 14; n_CDKN2Ap16_ = 43; n_RASSF1A_ = 11; n_TERT_ = 33; n_WT1_ = 18), and high grade tumor samples (G3-G4) (n_CDH1_ = 42; n_CDKN2Ap16_ = 92; n_RASSF1A_ = 30; n_TERT_ = 85; n_WT1_ = 21). A statistically significant increase of *TERT* methylation was found comparing tumor-free control to low-grade tumors (*p* = 0.022) and to high grade tumors (*p* = 0.021), the steady increase with tumor progression is a significant correlation (*p* = 0.003) Spearman correlation. *WT1* methylation decreased significantly comparing tumor-free control to low grade tumor samples (*p* = 0.043) Student’s t-test. (**C**) Methylation of target genes in the patient samples grouped by gender and age. A Spearman correlation analysis matrix showed no significant correlation between overall age and promoter methylation for any of the analyzed genes. * *p* < 0.05.

The methylation of CDH1, CDKN2Ap16, RASSF1A, TERT and WT1 were analyzed in 144 NSCLC and 7 healthy control tissue samples (a total of 151 patient samples), as summarized in Table 1. The analyses compare 4 female and 3 male tumor-free control samples (*n* = 7) to the tumor samples which we divided into low grade (G1 and G2) tumors (*n* = 48; 25 female and 23 male) and high grade (G3 and G4) tumors (*n* = 96; 31 female and 65 male). Due to their central role in diagnostics and therapy-design we also include EGFR and KRAS mutational status in the analyses, where in low-grade tumors, 42% of samples are mutated (15 KRAS mutations, 5 EGFR mutations) and in high-grade tumors 31% of samples are mutated (23 KRAS mutations, 5 EGFR mutations). Not all of the archival samples could be successfully and repeatedly sequenced in the target regions which led to missing data, shown as white cells in Table 1. The resulting datasets for the individual target genes are n_CDH1_ = 58; n_CDKN2Ap16_ = 142; n_RASSF1A_ = 42; n_TERT_ = 125 and n_WT1_ = 42. CDH1 methylation in all analyzed samples ranges from 1.8% to 19.0% promoter methylation, with no variation between individual groups. The promoter of the CDKN2Ap16 gene showed methylation levels ranging from 0.6% to 61.2%, with no differences between control (*n* = 7) the average wildtype (*n* = 89) and mutant (*n* = 46) tumor samples and between the female (*n* = 54) and male (*n* = 81) samples. RASSF1A promoter methylation ranged from 0.6% to 67.6%, with no clear differences among individual groups. (Figure 1A). TERT methylation was measured to be 1.2% to 76.6%, with an average methylation of 21% in females (*n* = 45) compared to 28.2% in male (*n* = 73) tumor samples (*p* = n.s.). Significantly lower methylation was recorded in the G0-tumor free control samples (14.4%; *n* = 7) as well as wildtype tumor samples (22.8%; *n* = 78) compared to EGFR/KRAS mutant samples (28.6%; *n*= 39) (*p* = 0.050 and *p* = 0.046; respectively) (Figure 1A). The broadest methylation spectrum was detected in WT1, ranging from 1.8% to 94.4%. Here, the average methylation of the tumor-free control (69.6%; *n* = 3) is higher, but without reaching significance due to low numbers, than the averages of female (36.7%; *n* = 18) and male (37.7%; *n* = 21) tumor samples as well as wildtype (37.7%; *n* = 23) and EGFR/KRAS mutated tumor samples (41.6%; *n* = 16). Figure 1B shows the different transformations of methylation levels throughout tumor grades. CDH1, CDKN2Ap16, and RASSF1A have comparable methylation values in tumor free control samples, low grade and high grade tumor samples. TERT showed a steady increase in actual methylation levels with an increasing tumor grade (*p* = 0.003, Spearman correlation). A significant increase (*p* = 0.05, student’s t-test) of TERT promoter methylation was detected in the tumor samples compared to the healthy controls, as well as between low and high-grade tumors (*p* = 0.010). For WT1, a significant (*p* = 0.043) decrease in methylation was recorded between tumor-free control samples (69.6%, *n* = 3) and low grade tumor samples (31.1%; *n* = 18). There is no significant difference between the WT1 methylation levels comparing low to high grade (42.6%; *n* = 21) tumor samples. Considering epigenetic aging and especially the pre/post-menopausal differences in methylation levels, we analyzed the promoter methylation levels in different age groups for both genders (Figure 1C). Although none of the analyzed differences reached statistical significance, the general trend of higher methylation in males was repeatedly observed. RASSF1A indicates an opposite trend, where, in younger females, it showed a distinctive methylation peak. 

It is known that smoking affects the DNA methylation in cells of different tissues of the human body. Therefore, an association of the methylation levels of the here described gene promoters with smoking behavior would be of interest. Unfortunately, the smoking behavior of the FFPE sample cohort as well as the cell line cohort has not been fully documented, preventing a meaningful analysis.

Next, we aimed to compare the methylation patterns of CDH1, CDKN2Ap16, RASSF1A, TERT, and WT1 in FFPE samples to cell culture models of NSCLC. We analyzed five female and nine male (14 cell lines in total), of which one female (IMR90) and one male (MRC-5) cell line were a benign lung control cell line. 

Since not all relevant molecular mutations are reported for the cell lines used here, we performed Next Generation Sequencing (Illumina, True Sight Tumor 15 Panel) to complete the cell line information given in Table 2, which we further expanded with the methylation information from in-house validated assays targeting the promoter regions of CDH1, CDKN2Ap16, RASSF1A, TERT, and WT1. IMR90 and MRC-5 cell lines represent non-tumor controls. The control cell lines IMR90 and MRC-5 show low methylation levels overall, which is in line with the reference methylome of IMR90 available via Encode (www.encodeproject.org). For the tumor cell lines, the methylation levels were generally low in CDH1 and CDKN2Ap16, where in the latter, H2347, Calu1, and H441 are contrasting with distinct high methylation levels. For RASSF1A, TERT, and WT1, the individual cell lines’ methylation levels span the whole range, with average methylation levels of RASSF1A at 29.0%, TERT at 34.8%, and WT1 at 60.1% all exceeding the levels recorded from the FFPE cohort. Statistical analyses show no significant interdependence among the methylation markers but individual correlations with EGFR and KRAS mutation. Here, a significant (*p* = 0.041) increase in methylation was found in CDKN2Ap16 when KRAS is mutated. When taking EGFR and KRAS into account, mutated cell lines showed no significant methylation change compared to the EGFR/KRAS wild-type cells (*p* = 0.175). Similarly, WT1 methylation is significantly increased (*p* = 0.037) in KRAS mutated cells. Comparing cell lines regarding their isolation site, a statistically significant (*p* = 0.004) difference in WT1 promoter methylation was found between cell lines stemming from primary versus metastatic tumors. The combined methylation score of the investigated epigenetic markers indicates that the EGFR- and KRAS-mutated samples are associated with hypermethylation in the screened cell lines. This agrees with the findings of Bjaanaes et al., who found, respectively, 436 versus 2 hypermethylated CpGs in the EGFR and KRAS mutated versus wildtype LC samples [50].

### 2.2. 2D vs. 3D Cell Culture and Methylation Level Stability

The clear difference between the promoter methylation observed in the cultured cell lines compared to the patient tumor samples may be explained in part by the artificial growth conditions for the cell lines in vitro. The prolonged culturing of cells on plastic dishes in a monolayer causes culture evolution and genetic drift [51]. Tumors usually form complex three-dimensional structures, which cannot be assumed to accurately be recapitulated by a cultured monolayer. Due to this fact, several 3D culture approaches have been established in recent years. In order to evaluate if methylation patterns change according to the cultivation method, cells were grown on non-adherent culture dishes to induce spheroid formation. Table 3 shows the difference in promoter methylation of the studied genes in the 2D vs. 3D cultured cell lines together with the methylation variability in 2D and 3D cell cultures over the analyzed CpG sites.

**Figure 2:**
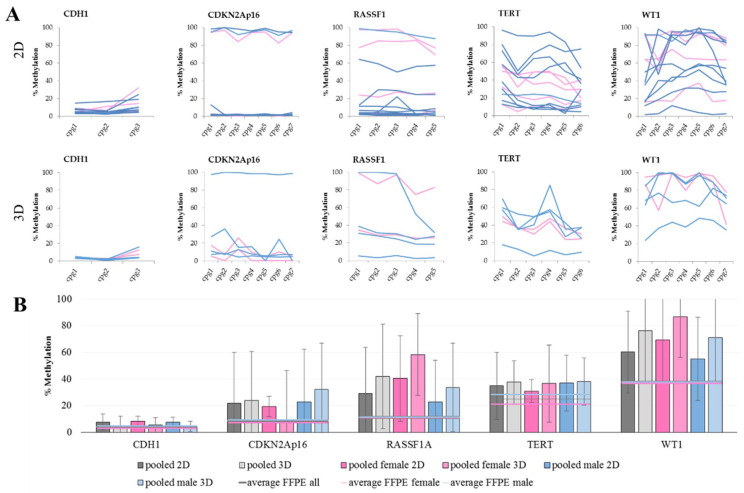
DNA methylation in cell lines. (**A**) The methylation levels over all analyzed CpG sites in 2D (top) and 3D (bottom) culture. Each line represents one female (pink) or male (blue) cell line. Within CGIs, the CpG sites show a fluctuation in methylation levels. This observation especially pertains to *RASSF1A, TERT*, and *WT1* in both culture conditions, as well as for *CDKN2Ap16* in 3D culture. (**B**) The average DNA methylation levels with standard deviation of grouped cell lines in the five marker genes analyzed in 2D and 3D cultures, shown as pooled values as well as pooled female and pooled male. Furthermore, average values of male, female, and all FFPE samples were added for reference.

When comparing the methylation levels of 2D cultures with 3D cultures, we see that some genes are more sensitive to the culture change than others. The methylation levels of *CDH1* and *TERT* are not changing with culture condition. This also seems to be the case for CDKN2Ap16, but when looking at the gender of the cells, an increase in male cell lines is observed, while the methylation levels in females are stable. An increase in methylation is also evident for *RASSF1* and *WT1*. These higher DNA methylation levels are an already described but not thoroughly understood phenomenon [52,53,54]. Considering the lower methylation levels in FFPE samples (Figure 2B), we opted for a traditional 2D cell culture (i.e., due to its lower cost and superior ease of use).

The average methylation of single CpG sites within one CGI indicates high fluctuation, especially for *TERT* and *WT1*, which is also reflected in the high standard deviations of the average methylation over all available CpG sites.

### 2.3. EC_50_ Values

**Figure 3:**
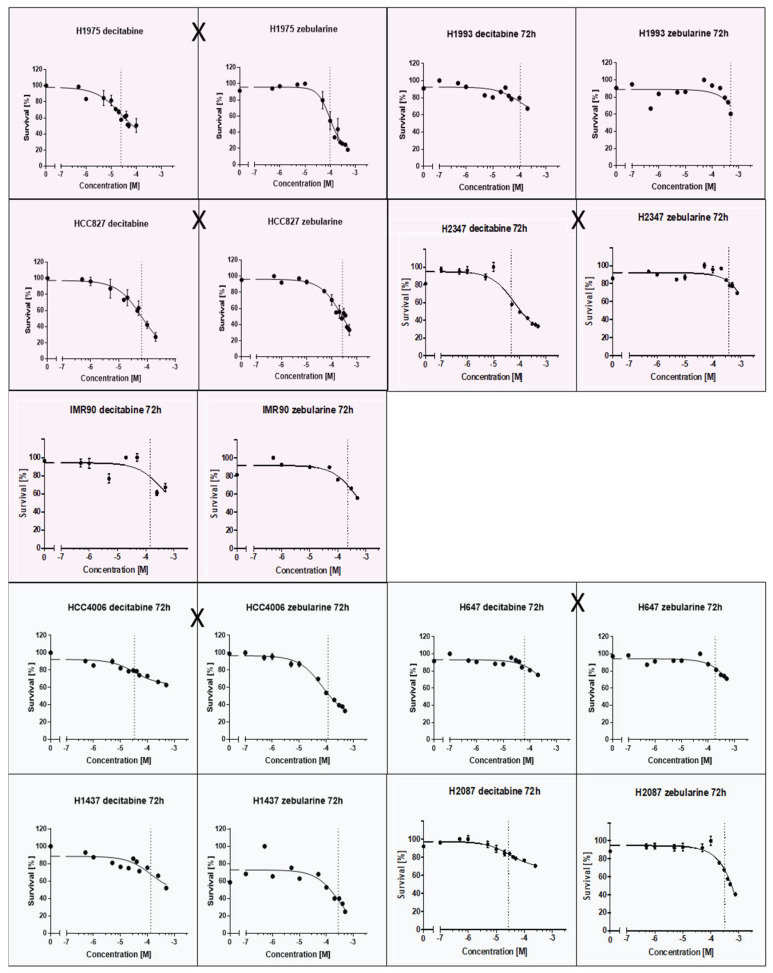
Dose-response curves of NSCLC-cell lines treated with the DNMTIs decitabine and zebularine; shading discriminates male (blue) and female (pink) cell lines. *EGFR/KRAS* mutation is indicated by an X between the two curves of a cell line.

**Figure 4:**
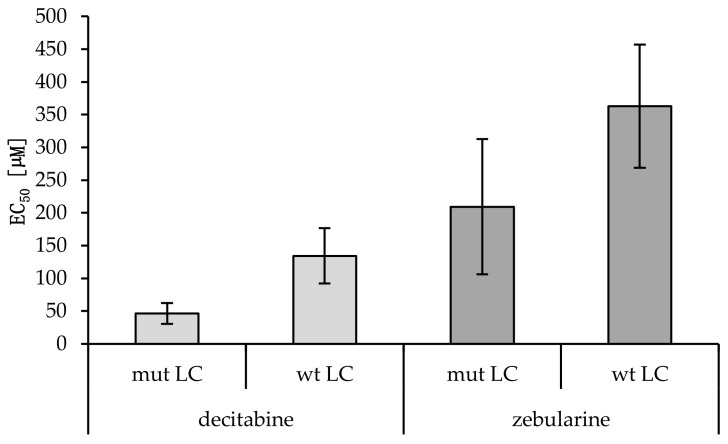
EC_50_ values of wild-type versus mutant cancerous cell lines treated with decitabine or zebularine. Averages and standard deviations are shown (n_mut LC_ = 5; n_wt LC_ = 3). A statistically significant difference between the decitabine EC_50_ values of mut versus wt cell lines was found (*p* = 0.01, Student’s t-test). No statistical significance was reached for zebularine (*p* = 0.12; Students t-test).

A putative relation between *EGFR/KRAS* mutation and the promoter methylation of *CDH1*, *CDKN2Ap16*, *RASSF1A*, *TERT*, and *WT1* could influence tumor progression and therapy response. TKIs and methyltransferase inhibitors (DNMTI) are two classes of cancer therapeutics. We treated several different cell lines with TKIs and DNMTIs, evaluating their respective sensitivity and EC_50_ values, in connection with the prevalent promoter DNA methylation levels. In this way, we could evaluate any predictive potential of the epigenetic markers. Figure 3 shows the dose–response curves for decitabine or zebularine; for the DNMTI-treated NSCLC cell lines, the dashed lines indicate EC_50_ concentrations. The TKI response curves were published by Jacobi et al. [55]. Both treatment strategies are summarized in Table 4, compared to the cell lines’ individual methylation scores and mutational statuses. Despite the small sample size, a trend of higher DNMTI sensitivity in the *EGFR/KRAS* mutated cell lines compared to the wildtype cell lines (excluding the benign cell line IMC90) is observable (Figure 4). This is significant for the treatment with decitabine (*p* = 0.01). No significant correlations exist between DNMTI treatment and sample gender.

## 3. Discussion

Promoter methylation of the genes *CDH1, CDKN2Ap16, RASSF1A, TERT*, and *WT1* was analyzed using optimized and validated pyrosequencing assays in 14 cell lines and 151 NSCLC tissue samples, as summarized in Table 1 and Table 2.

The methylation ranges observed in the tumor samples differed between genes, with low methylation ranges for *CDH1*, similar to the observed range in the NSCLC cell lines. The highest range was observed in *WT1* followed by *TERT*, *RASSF1A*, and *CDKN2Ap16*. The latter showed an almost all-or-nothing methylation behavior in cell lines, which is suggested to interfere with the expression of thep16 protein and the related cell cycle control. This could also be the case in the tissue samples, where methylation levels are evaluated for a cell mixture rather than exclusive tumor cells and will be investigated further. The hypermethylation of the *CDKN2Ap16* promoter has been linked to an early stage in the pathogenesis of LC [33]. Furthermore, *CDKN2Ap16* and *RASSF1A* methylation have been associated with recurrence following the resection of stage I NSCLC [56].

In tissue samples, we could show the statistically significant steady increase in *TERT* methylation levels with increasing tumor grades (*p* = 0.05, Students t-test; *p* = 0.003, Spearman correlation).

A putative correlation between promoter methylation levels and common mutations in NSCLC, specifically *EGFR* and *KRAS*, was evaluated in tissue samples and cell lines. Even though the genetic and epigenetic markers are not closely related, considering the merged methylation score, a trend towards higher methylation in the *EGFR* mutated cell culture samples, as well as a stronger trend in the *KRAS* mutated cell cultures, was observed (Table 2). These results align with the findings of other studies [50,57]. In our FFPE cohort, a significantly higher methylation was only recorded for the *TERT* gene. Interpretation of trends for the other genes would be speculative, also because the mutated samples were underrepresented in our cohort.

Figure 2 reveals that in all analyzed genes, the methylation levels exceed those observed in tissue samples. In an attempt to explain the observed differences between patient samples and cell lines, a 3D cell culture approach that mirrors in vivo tumor conditions more closely was tested [55]. Unfortunately, this approach did not yield a more accurate representation of patient data (Table 3).

This all makes a point that in epigenetic research, the value of tissue samples is superior to cell cultures, as the epigenetic nature of DNA methylation is influenced by dynamic factors as age and lifestyle. Figure 1C summarizes the methylation levels of *CDH1/CDKN2Ap16/RASSF1A/TERT* and *WT1* over different age groups. Here, specifically *RASSF1A*, and other genes by trend, show an increase in promoter methylation in older males. Unfortunately, we do not have information on smoking behavior of the patients, which would be an interesting lifestyle-representing and important risk factor to be analyzed.

In vitro analyses are the basis for modern precision medicine and are therefore also key in epigenetic research. We evaluated the treatment of cell lines with the DNMTIs decitabine and zebularine, showing, on average, comparable sensitivities of the male cell lines and the female cell lines (dec 54.6 vs. 104.0 and zeb 309.4 vs. 224.4 µM EC_50_), as shown in Table 4. A more pronounced difference was observed in the mutated versus wildtype samples (dec 46.3 vs. 134.4; *p* = 0.003 and zeb 209.2 vs. 363.1 µM EC_50_; *p* = 0.163). A lower methylation score (< 8) indicates higher sensitivity to decitabine (76.3 vs. 81.1 µM EC_50_) but lower sensitivity to zebularine (192.6 vs. 311.5 µM EC_50_), though with both treatments toxicity was higher in cells with high methylation scores.

The formation of metastases is of major concern in the treatment of LC, as well as other cancer entities. The metastatic potential of NSCLC is influenced by intrinsic and extrinsic cell microenvironmental factors but has also been associated with EMT. The reduction of *CDH1* expression is thought to promote metastasis and is an established indicator for a poor prognosis in a variety of tumor entities [58,59,60]. There is a putative epigenetic link between *CDH1* and one of its transcription factors, *WT1*. *WT1* can directly suppress the expression of *CDH1* by binding its promoter, thereby enhancing invasion [61]. This phenomenon has been reported in NSCLC and prostate cancer cell lines [61,62]. Both involved genes possess CGI-overlapping promoter regions and are reportedly prone to epigenetic regulation. Our research data confirm their tumor-associated differential methylation.

## 4. Materials and Methods

### 4.1. Assay Development and Validation

Pyrosequencing-based assays were developed and validated for clinical use to detect the DNA promoter methylation of five genes.

For determination of each assay’s Limit of Blank (LoB), the sequencing results of at least fifty no template control (NTC) runs were evaluated in each CpG site. The LoB values equal the average plus 1645 times the standard deviation in relative fluorescence units (RFU) [63]. Limit of Quantification (LoQ) indicates the value above which a measurement can be evaluated quantitatively. For this, the average reading of the NTC samples was increased tenfold. This approach was preferred over the common method of adding the average NTC measurement to the ten-fold standard deviation, since the tenfold averages were always higher, thereby ensuring greater assay certainty. The Limit of Detection (LoD) is the lowest concentration of the analyte (the bisulfite-converted DNA used) at which correct sequence determination is still possible. For this purpose, we determined the lowest analyte concentration at which the respective test reproduces a stable signal, i.e., methylation levels in the range of average methylation with 10 ng PCR input ± 2%. LoD values are given as the bisulfite-treated-DNA concentration to be used as the PCR template. LoD determinations were performed only with methylated, bisulfite-converted DNA (Promega, Methylated Human Control N1231) and subsequently checked for DNA from the cell lines and FFPE tissue.

The validation results for each gene of interest are summarized in Table 5.

### 4.2. Sequencing Assays

The genes *CDH1, CDKN2Ap16, RASSF1A, TERT*, and *WT1* were selected based on their roles as biomarkers in NSCLC and their putative gender sensitivity based on the current literature and our own research findings (reviewed in [64]). Among the selected markers, *RASSF1A* [65,66,67,68,69,70,71,72] and, to a lesser extent, *CDKN2Ap16* [73,74,75,76,77] are already accepted as methylation markers. The applicability of *CDH1* [78,79,80,81,82,83] and *WT1* [45,61,84,85] is still under investigation but may have a broader impact on many cancer entities; their interdependence adds to this potential. *TERT* [86,87] methylation is mainly reported in combination with other genes in tumor-associated epigenetic signatures [88,89,90]. Pyrosequencing became the method of choice for this work due to its simple translation to clinical routines and the comprehensive availability of the applicable technical equipment in contemporary pathology laboratories. Further design criteria were (1) intersection of the sequencing assay with the promoter region and CGI on the DNA; (2) a PCR amplicon length of approximately 100 bp up to a maximum of 300 bp to avoid running into constraints due to formalin-induced DNA fragmentation; (3) a sequencing region that covers at least two CpG sites. Based on these criteria, the software PyroMark Assay Design 2.0 (Qiagen, Hilden, Germany) was used to create at least three different assays per gene. An assay comprises a set of three primers (two PCR primers and an additional sequencing primer). Out of the three designed assays, one was chosen based on offering the best performance in the initial PCR and sequencing runs (Table 6). For the final primer sets, the PCR parameters were optimized. The time and temperature of the primer annealing and extension steps, as well as the cycle numbers, were adjusted, and the addition of the PCR additives MgCl2, Betain, and CoralLoad (Qiagen) was tested to provide one single well-defined PCR product in the melting curve analysis or agarose gel electrophoresis. The final PCR settings are summarized in Table 7. The validation of sequencing assays was performed on the DNA samples derived from cell lines and was confirmed using the DNA samples isolated from formalin-fixed paraffin-embedded (FFPE) tissue specimens. The validation strategy was based on the Clinical and Laboratory Standards Institute (CLSI) approved guideline EP17-A [63] and included determination of the values for the LoB, LoQ, and LoD (Table 5).

### 4.3. Formalin-Fixed, Paraffin-Embedded (FFPE) Samples

FFPE specimens of LC patients were obtained from Pathologylab Zams, Tyrol, according to the positive ethics vote EK GZ 08/2015–2018 issued by the Danube University Krems ethics commission. The study cohort comprised 151 cases, with a 40:60 female:male gender ratio. The average age was 67 years. Females averaged 64.3 years old, which was slightly younger than the male patients, who averaged 68.6 years. The FFPE cohort was characterized by their *EGFR* and *KRAS* mutational status, *EGFR* copy number variation, and *ALK* and *ROS1* gene rearrangement (FISH Analysis applying a Vysis *ALK* Break Apart FISH Probe (Abbott GmbH, Wiesbaden, Germany); *ROS1*: ZytoLight SPEC *ROS1* Dual Color Break Apart Probe (ZytoVision GmbH, Bremerhaven, Germany)). The overall mutation frequency in the FFPE cohort was 40%, with *KRAS* mutations (*n* = 39) being the most frequent mutational event, followed by *EGFR* mutations (*n* = 10) and gene amplification (*n* = 6). In the entire cohort, only 3 *ALK* rearrangements and no *ROS1* rearrangements were identified. All cancerous tissue samples were collected before the onset of neoadjuvant therapy. Control tissue were selected based on histological assessment

The extraction of DNA was carried out using a QiaAmp^®^ DNA FFPE Tissue Kit (56404, Qiagen) according to the manufacturer’s instructions, automated on a QiaCube (Qiagen, Hilden, Germany). After fluorimetric determination of the sample concentration, 1 µg DNA was bisulfite-converted for subsequent methylation analysis.

### 4.4. Cell Culture

The following LC cell lines were obtained from ATCC: NCI-H1975 (ATCC^®^ CRL-5908^™^), HCC827 (*ATCC*^®^ CRL-2868^™^), IMR90 (*ATCC*^®^ CCL-186^table^), NCI-H2347 (*ATCC*^®^ CRL-5942^™^), NCI-H2087 (*ATCC*^®^ CRL-5922^™^), NCI-H1993 (*ATCC*^®^ CRL-5909^™^), NCI-H647 (*ATCC*^®^ CRL-5834^™^), NCI-H1437 (*ATCC*^®^ CRL-5872^™^), and HCC4006 ((*ATCC*^®^ CRL-2871^™^). All cell lines, with the exception of IMR90, were grown in an RPMI-1640 medium (31870-025, Thermo Fisher Scientific, Waltham, MA USA) supplemented with 10% FBS (10270106 ThermoFisher Scientific), 1% Penicillin/Streptomycin (15140122, ThermoFisher Scientific), and 2 mM Glutamine (25030081, ThermoFisher Scientific). IMR90 and MRC-5 cells were cultured in an MEM α medium (22561-021 ThermoFisher Scientific) containing 10% FBS, 1% Penicillin/Streptomycin, and 2 mM Glutamine. IMR90 and MRC-5 cell lines represent non-tumor controls. MRC-5 cells were donated from Prof. R. Klein (IMC Krems). Cells were kept at 37 °C in a humid atmosphere with 5% CO_2_. Cells were split 1:4 using Trypsin-EDTA (0.25%, 25200056, ThermoFisher Scientific) when 70% confluent. For 3D cultures, 100,000 cells/well were seeded in low attachment 6-well plates (Thermo Scientific™, Nunclon Sphera, 174944) and cultured in standard conditions. Spheroids were harvested after nine days.

DNA isolation from the cell line pellets was performed using the QIAmp^®^ DNA Mini Kit (51304, Qiagen).

For all cell lines without *KRAS* and *EGFR* mutational statuses published by ATCC, an NGS analysis was performed using the Illumina True Sight Tumor 15 gene panel on an Illumina MiniSeq system according to the manufacturer’s instructions and 5% allele frequency limits. The assay covered the hotspot regions of 15 tumor-relevant genes, including *AKT, BRAF, EGFR, ERBB2, FOXL2, GNA11, GNAQ, KIT, KRAS, MET, NRAS, PDGFRA, PIK3CA, RET*, and *TP53*. The mutational statuses of *EGFR* and *KRAS* are reported in Table 2. The following mutations were found in the other genes: H1993 had an amplified *MET* gene and *TP53* SNP 726C > G; HCC827 was mutated in *TP53* (648G > T); H1437 harbored two *TP53* SNPs: 1175A > C and 800G > C, as well as a heterozygous intronic *ERBB2* deletion; H2087 was mutated in the genes *NRAS* (181C > A), *BRAF* (1789C > G), and *TP53* (469G > T); H647 was also mutated in *TP53* (c.782 + 1G > T); and HCC2935 showed two *TP53* mutations (818G > A and 673-36G > C).

### 4.5. Zebularine/Decitabine Treatment and EC_50_ Determination

In order to get an insight into the sensitivity of cell lines to DNMTI treatment, we selected H1975 (female KRAS/EGFR mutated), HCC827 (female KRAS/EGFR mutated), HCC4006 (male KRAS/EGFR mutated), and H1437 (male- KRAS/EGFR wild-type) cells as earlier their responsiveness to TKI was intensely investigated ([55]). With gender influence being one of our major research questions, we selected in addition H1993 and H2347 as female and H647 and H2087 as additional male cell lines. IMR90 cells are analyzed as benign control cells (female).

NCI-H1975, HCC827, NCI-H1993, NCI-H2347, NCI-H2087, NCI-H647, IMR90, HCC4006, NCI-H1437, and A549 cells were seeded as 1 × 10^4^ cells /100 µL, 1.5 × 10^4^ cells /100 µL, 1 × 10^4^ cells /100 µL, 1.5 × 10^4^ cells /100 µL, 1 × 10^4^ cells /100 µL, 1.5 × 10^4^ cells /100 µL, 0.5 × 10^4^ cells /100 µL, 1 × 10^4^ cells/100 µL, 1 × 10^4^ cells /100 µL, and 1 × 10^4^ cells /100 µL, respectively, in 96-well plates and left to adhere for 24 h. Subsequently, the cells were treated with zebularine at concentrations of 0–500 µM or decitabine at concentrations of 0–500 µM for 72 h. The cells were then harvested for promoter DNA methylation analysis or used for EC_50_ determination using the WST-1 reagent (11644807001, SigmaAldrich, St.Louis, MO, USA) according to the manufacturer’s protocol. The EC_50_ values were determined using GraphPad Prism version 7.03 for Windows, GraphPad Software, La Jolla California USA, www.graphpad.com.

For determination of the viability, reduction cells were seeded alike and treated for 72h with 6 different doses of zebularine or decitabine ranging from 0µM to the fifth fold of the respective EC50 value. Harvested cells were stained with WST-1 reagent and analyzed in a SpectraMax i3x Multimode reader (Molecular Devices, LLC; San Jose, CA, USA).

### 4.6. Statistical Methods

All methylation levels are given in % or score. The percentage methylated is the average methylation level of all analyzed CpG sites within the analyzed CGI / gene. The number of CpG sites per gene is shown inTA2A. Student’s t-tests were performed in Microsoft Excel 2010.

Spearman correlation matrices and Mann–Whitney tests were generated in GraphPad Prism version 5 for Windows, GraphPad Software, La Jolla California USA, www.graphpad.com.

The data analysis for the TKI section was generated using the Real Statistics Resource Pack software (Release 6.8) Copyright (2013–2020) Charles Zaiontz. www.real-statistics.com. For DNMTI treatment analyses, Dunnett’s test, Tukey’s test, and two-tailed Student’s t-test were applied.

The EC_50_ values were determined using GraphPad Prism version 7.03 for Windows, GraphPad Software, La Jolla California USA, www.graphpad.com.

## 5. Conclusions

In conclusion, we showed that patient gender is a long-overlooked, yet crucial, element of personalized medicine. We found that the DNA methylation markers specifically differed between sexes in the NSCLC tissue samples. Furthermore, we established validated pyrosequencing-based assays for the determination of promoter methylation in five genes.

## Figures and Tables

**Table 1:** Methylation patterns of formalin-fixed paraffin-embedded (FFPE) samples along with KRAS and epidermal growth factor receptor (EGFR) mutational status, gender, and tumor grade; mutated EGFR and KRAS are indicated by darker grey shadowing, pink and blue represent patient gender as female or male, respectively; as for the methylation levels of CDH1, CDKN2Ap16, RASSF1A, TERT, and WT1, the shown values are average methylation scores (%) over the analyzed CpG-island (CGI) in at least 3 technical replicates. Light green indicates low methylation (<21%), bright yellow indicates strong methylation (>59%), and median methylation is represented by a yellow-green color. Blank positions are missing data, as not all analyses were successful in 3 replications on limited sample volumes. A combined methylation score was calculated summing the ordinal methylation levels, for which “low” methylation counts for 1, “med” for 2, and “high” methylation for 3; considering missing values, the methylation score was divided by 5, ranging from 0 to 3, it allows a very rough interpretation of data trends, where differences only occur in high-grade tumors, where the average methylation scores of male and mutated samples are higher than the average.

	Mutations		Methylation [%]					
FFPE Sample Tumorgrade gender	EGFR	KRAS	CDH1	CDKN2Ap16	RASSF1A	TERT	WT1	Combined Methylation Score
G0 f	wt	Q61H	3.5	1.7	6.9	10.5	94.4	1.4
G0 f	wt	wt	2.2	49.5		14.3		1.3
G0 f	wt	wt		17.3		10.8		1.0
G0 f	wt	wt		6.9		21.7		1.5
G0 m	wt	wt		2.3		4.6	29.5	1.3
G0 m	wt	wt		14.3		26.9		1.5
G0 m	wt	wt		3.9		11.7	84.8	1.7
G1 f	wt	wt		7.2	67.6	22.8	1.8	1.8
G1 m	wt	G12V			11.9	37.7		1.5
G1 m	wt	wt		20.5		37.1		1.5
G2 f	G719C; S768I	wt		7.3		13.8		1.0
G2 f	E746_A750del (#)	wt		42.9		38.6		2.0
G2 f	L858R	wt	2.2	2.4				1.0
G2 f	L861Q	wt	2.3	1.9		17.2		1.0
G2 f	wt	G12C		6.3			4.1	1.0
G2 f	wt	G12C				12.8		1.0
G2 f	wt	G12C		5.7		23.5		1.5
G2 f	wt	G12C	3.2	5.0	1.7	33.2	22.5	1.4
G2 f	wt	G12D		3.0				1.0
G2 f	wt	G12F		0.9			86.9	2.0
G2 f	wt	G12V		3.0			6.1	1.0
G2 f	wt	wt		3.1		5.3		1.0
G2 f	wt	wt		1.7		14.5	62.3	1.7
G2 f	wt	wt		46.6		40.3		2.0
G2 f	wt	wt		1.5		17.6		1.0
G2 f	wt	wt		4.1		25.3		1.5
G2 f	wt	wt		3.3		5.9	90.2	1.7
G2 f	wt	wt		2.1				1.0
G2 f	wt	wt		8.5		7.6		1.0
G2 f	wt			4.5				1.0
G2 f	wt	wt		6.9		7.1	6.5	1.0
G2 f	wt	wt	2.2	0.6	1.2	3.8	17.5	1.0
G2 f	wt	wt	2.5	1.7	6.0	21.9	24.3	1.4
G2 f	wt	wt	4.2	6.3		20.7	60.6	1.5
G2 m	P733L	wt		1.3				1.0
G2 m	wt	G12C	2.0	8.6	6.5	7.9	35.9	1.2
G2 m	wt	G12D		1.2			7.9	1.0
G2 m	wt	G12D	19.0	6.6	5.0	4.2	13.9	1.0
G2 m	wt	G12D	8.8	3.3	6.2	16.0	44.1	1.2
G2 m	wt	G12V	2.2	3.3		5.5		1.0
G2 m	wt	G12V		38.6	45.3	40.4		2.0
G2 m	wt	G12V	3.3	22.1	2.8	27.8		1.5
G2 m	wt	wt		61.2		56.9		3.0
G2 m	wt	wt		2.5		10.9	26.1	1.3
G2 m	wt	wt		1.0				1.0
G2 m	wt	wt	2.5	2.7		16.8		1.0
G2 m	wt	wt						0.0
G2 m	wt	wt	2.3	3.1				1.0
G2 m	wt	wt		29.0		29.3		2.0
G2 m	wt	wt	3.2	1.9	10.8		13.4	1.0
G2 m	wt	wt						0.0
G2 m	wt	wt		5.0		1.2		1.0
G2 m	wt	wt		0.9		34.2		1.5
G2 m	wt	wt		3.4		1.8	36.0	1.3
G3 f	E709K; L858R	wt		3.5		51.5		2.0
G3 f	E746_#	wt	4.5	3.8	5.4	6.6	21.9	1.2
G3 f	T790M; T751ind	wt	3.0	2.9	1.7	38.8		1.3
G3 f	wt	G12A		8.1		15.0	9.8	1.0
G3 f	wt	G12C		0.9		28.6	59.5	2.0
G3 f	wt	G12C		2.4		44.5		1.5
G3 f	wt	G12C	2.7	3.3	1.7	7.6		1.0
G3 f	wt	G12C	3.0	17.4	0.6	22.6		1.3
G3 f	wt	G12V		1.8		46.8		1.5
G3 f	wt	wt		10.1	2.7	40.1		1.0
G3 f	wt	wt				13.3		1.0
G3 f	wt	wt		0.9		2.4		1.0
G3 f	wt	wt		2.2		18.7		1.0
G3 f	wt	wt		1.9		22.0		1.5
G3 f	wt	wt		2.2		12.0		1.0
G3 f	wt	wt		3.5		8.8		1.0
G3 f	wt	wt		1.1				1.0
G3 f	wt	wt		16.4				1.0
G3 f	wt	wt	1.8	8.7		10.3		1.0
G3 f	wt	wt	2.5	2.8				1.0
G3 f	wt	wt	1.8	4.1		10.4		1.0
G3 f	wt	wt		5.6		20.6		1.0
G3 f	wt	wt		18.4		8.4	27.4	1.3
G3 f	wt	wt		15.3			30.2	1.5
G3 f	wt	wt	7.2	37.6	31.8	71.6	37.1	2.0
G3 f	wt	wt	2.3	5.9	3.7	9.8		1.0
G3 f	wt	wt	2.2	2.0		4.5		1.0
G3 f	wt	wt		6.9		36.9		1.5
G3 f	wt	wt		4.5		24.4		1.5
G3 f	wt	wt	2.3	3.6		16.8		1.0
G3 f	wt	wt		19.4	6.9	21.7	92.5	1.8
G3 m	E746K	wt	3.7	11.1	2.4	27.1		1.3
G3 m	L858R	wt		1.1	9.9	38.8		1.3
G3 m	wt	G12A		2.6		25.8		1.5
G3 m	wt	G12A	2.0	1.9		42.0		1.3
G3 m	wt	G12A		9.6		27.1		1.5
G3 m	wt	G12C		24.7	3.2	35.2		1.7
G3 m	wt	G12C	1.8	2.5				1.0
G3 m	wt	G12C		13.0		56.2		2.0
G3 m	wt	G12C		5.0	5.3	35.4	44.4	1.5
G3 m	wt	G12C	2.2	1.7	2.8	5.4	11.5	1.0
G3 m	wt	G12C	6.5	48.8		21.3		1.7
G3 m	wt	G12D		1.3		54.3		2.0
G3 m	wt	G12D		2.9		34.3		1.5
G3 m	wt	G12V	2.7	5.9		28.9	93.4	1.8
G3 m	wt	G12V	2.2	2.4	30.1	19.4		1.3
G3 m	wt	G13C	2.7	6.1		37.2		1.3
G3 m	wt	G13C		4.8	25.5	27.5	63.4	2.0
G3 m	wt	G13C	4.2	4.6		39.1	86.8	1.8
G3 m	wt	G13D	7.7	32.2	4.3	25.7		1.5
G3 m	wt	wt	12.8	2.0		19.2		1.0
G3 m	wt	wt	11.3	24.9		37.6		1.7
G3 m	wt	wt						0.0
G3 m	wt	wt		4.0		23.2		1.5
G3 m	wt	wt		3.3	1.4	3.6		1.0
G3 m	wt	wt		33.2	3.0	16.0		1.3
G3 m	wt	wt		1.5		49.2		1.5
G3 m	wt	wt						0.0
G3 m	wt	wt		5.1		36.9		1.5
G3 m	wt	wt		8.6		34.4	87.0	2.0
G3 m	wt	wt		4.2		27.2	19.9	1.7
G3 m	wt	wt		5.8		30.4		1.5
G3 m	wt	wt		3.6		8.3		1.0
G3 m	wt	wt	4.2	0.9				1.0
G3 m	wt	wt	3.5	2.4		76.6		1.7
G3 m	wt	wt				1.6		1.0
G3 m	wt	wt		4.4		22.0		1.5
G3 m	wt	wt		28.6		15.6		1.5
G3 m	wt	wt		8.0				1.0
G3 m	wt	wt	10.2	16.4	20.3	8.4	7.2	1.0
G3 m	wt	wt	3.0	3.1	24.8	20.1		1.3
G3 m	wt	wt	2.7	17.1	48.9	24.3		1.5
G3 m	wt	wt		13.5			8.9	1.0
G3 m	wt	wt	2.3	3.6	1.0	5.5	7.2	1.0
G3 m	wt	wt	17.2	3.2	2.9	13.2		1.0
G3 m	wt	wt	2.0	14.8	6.5	38.3		1.3
G3 m	wt	wt		1.1				1.0
G3 m	wt	wt	2.2	5.6		26.3		1.3
G3 m	wt	wt		1.1		12.1		1.0
G3 m	wt	wt	2.0	5.5		39.9	7.2	1.3
G3 m	wt	wt	2.0	3.4		63.2		1.7
G3 m	wt	wt		15.3		25.9		1.5
G3 m	wt	wt		15.8		26.9		1.5
G3 m	wt	wt		2.0		35.3		1.5
G3 m	wt	wt	3.8	2.1	3.7	36.0		1.3
G3 m	wt	wt		4.1		24.4		1.5
G3 m	wt	wt	2.2	1.6		45.8		1.3
G3 m	wt	wt	3.2	1.3	6.8	38.0		1.3
G3 m	wt	wt	3.2	8.0		22.9		1.3
G3 m	wt	wt	2.0	8.0		21.7		1.3
G3 m	wt	wt	3.2	10.1		21.8		1.3
G3 m	wt	wt		3.4		46.0	48.9	1.7
G3 m	wt	wt	2.7	5.9	6.8	60.1	57.4	1.8
G3 m	wt	wt		2.3	3.7	18.5	72.2	1.8
G4 m	wt	wt		3.0	18.2	30.3		1.3
G4 m	wt	wt	3.3	25.0	3.7	47.6		1.5

**Table 2:** Cell lines with the relevant information on gender, *EGFR*, and *KRAS* mutational status, as well as methylation levels for *CDH1. CDKN2Ap16. RASSF1A, TERT*, and *WT1*. f—female (pink shading); m—male (blue shading); MUT—mutated (dark grey); WT—wildtype (light grey); methylation levels under 21 % were scored as low—light green shading, those between 21 % and 59 % as medium—bright green shading, and levels above 59 % as high—yellow shading.

				Mutations		Methylation [%]					Combined Methylation Score ^a,b,c,d^
Cell Line	Gender	Source Site		EGFR	KRAS	CDH1	CDKN2Ap16	RASSF1A	TERT	WT1	
H1975	f	primary		MUT (L858R)	WT	15.8	1.4	24.7	43.5	88.0	**9**
H1993	f	metastasis		WT amplified	WT	5.2	1.6	80.3	21.3	22.0	**9**
HCC827	f	primary		MUT (Glu746_Ala750del)	WT	10.2	1.1	91.4	37.9	79.9	**10**
H2347	f	primary		WT	MUT (L19F)	3.8	91.6	2.4	43.8	91.3	**10**
IMR90	f	benign ctrl		WT	WT	5.7	1.2	3.4	9.1	65.9	**7**
HCC2935	m	primary		MUT (E746_T751 del, S752I)	WT	3.5	1.9	24.2	48.9	83.9	**9**
HCC4006	m	metastasis		MUT (L747_E749 del, A750P)	WT	12.7	1.0	5.9	8.3	25.1	**6**
Calu1	m	metastasis		WT	MUT (G12C)	7.0	97.2	9.4	56.7	80.1	**10**
Calu3	m	metastasis		WT	MUT (G13D)	4.5	1.6	3.6	71.3	73.9	**9**
H441	m	primary		WT	MUT (G12V)	6.2	95.3	93.6	21.9	91.1	**12**
H647	m	metastasis		WT	MUT (G12L)	3.7	1.6	1.4	12.0	37.9	**6**
H1437	m	metastasis		WT	WT	7.0	2.1	2.2	11.7	45.1	**6**
H2087	m	metastasis		WT	WT	3.8	1.1	57.3	84.3	52.9	**9**
MRC-5	m	benign ctrl		WT	WT	16.8	3.4	6.5	18.3	4.9	**5**

^a^ A combined methylation score was introduced summing the ordinal methylation levels, for which “low” methylation counts for 1, “med” for 2, and “high” methylation for 3. The resulting score ranges from 5 to 15. ^b^ female vs male cell lines, *p* = 0.338. Mann–Whitney test. ^c^ primary vs metastatic isolation site, *p* = 0.062. Mann–Whitney test. ^d^ EGFR/KRAS mutated vs WT cell lines, *p* = 0.09. Mann–Whitney test.

**Table 3:** Difference in promoter methylation in the cell lines cultured in 2D versus 3D methlyation increased significantly in HCC827 cells in the gene *CDNKA2p16* (*p* = 0.005), in HCC2935 cells in the genes *CDNKA2^p16^* (*p* = 0.000) and *RASSF1A* (*p* = 0.008), in HCC4006 cells in the genes *CDNKA2p16* (*p* = 0.000), *RASSF1A* (*p* = 0.000), *TERT* (*p* = 0.000), and *WT1* (*p* = 0.000), in Calu1 cells in the genes *CDH1* (*p* = 0.044) and *RASSF1A* (*p* = 0.044), in H1437 cells in the genes *CDNKA2^p16^* (*p* = 0.014) and. *WT1* (*p* = 0.049). f—female (pink shading); m—male (blue shading); methylation levels under 21% were scored as low—light green shading, those between 21% and 59% as medium—bright green shading, and levels above 59% as high—yellow shading.

Cell Line	Gender	2D CDH1	3D CDH1	2D CDKN2Ap16	3D CDKN2Ap16	2D RASSF1A	3D RASSF1A	2D TERT	3D TERT	2D WT1	3D WT1
H1975	f	15.8	4.8	1.4	2.6	24.7	28.7	43.5	39.2	88.0	79.3
HCC827	f	10.2	6.2	1.1	11.2	91.4	88.1	37.9	33.9	79.9	94.1
HCC2935	m	3.5	2.8	1.9	8.1	24.2	76.4	48.9	59.5	83.9	71.2
HCC4006	m	12.7	7.0	1.0	5.5	5.9	30.4	8.3	47.8	25.1	89.6
Calu1	m	7.0	3.8	97.2	98.4	9.4	33.7	56.7	43.5	80.1	84.3
H1437	m	7.0	3.7	2.1	16.9	2.1	3.9	2.1	10.7	2.1	39.1

**Table 4:** Summary of the EC_50_ values and viability reductions achieved for the lung cancer (LC) (IMR90 is benign) cell lines treated with the DNA methyl-transferase inhibitors (DNMTIs) decitabine and zebularine. For H1975, HCC827, HCC4006, and H1437, additional EC_50_ values for tyrosine kinase inhibitor (TKI) treatments with gefitinib and erlotinib are shown. Student’s t-test gave no statistically significant *p*-value comparing viability reduction and EC_50_ values for female vs. male cancerous cell lines (decitabine: *p* = 0.22, zebularine: *p* = 0.41). A statistically significant difference between the decitabine EC_50_ values of mut versus wt cell lines was found (*p* = 0.01). No statistical significance was reached for zebularine (*p* = 0.12) (Figure 4). A Mann–Whitney U test for the EC_50_ values of cancerous cell lines with methylations scores >8 compared to cancerous cell lines with methylation scores <8 showed no statistically significant difference for neither decitabine (*p* = 1.0) nor zebularine (*p* = 0.39).

Cell Line	Mutational Status	Methylation Score	EC_50_ Decitabine [µM]	EC_50_ Zebularine [µM]	Viability Reduction Decitabine	Viability Reduction Zebularine	EC_50_ Gefitinib	EC_50_ Erlotinib
H1975	EGFR mut	9	24.42	97.68	60%	42%	>10 µM	>10 µM
H1993	EGFR amp	9	83.5	493.8	36%	40%		
HCC827	EGFR mut	10	63.72	267.9	37%	62%	2.39 nM	1.29 nM
H2347	KRAS mut	10	46.85	378.1	34%	21%		
IMR90	wt	7	146.8	230.3	58%	54%		
H4006	EGFR mut	6	33	117.9	36%	77%	276 nM	259 nM
H647	KRAS mut	6	63.43	184.3	55%	33%		
H1437	wt	6	132.6	275.6	41%	21%	>10 µM	>10 µM
H2087	wt	9	187	319.9	32%	39%		
average female LC ^a^			54.6	309.4	42%	41%		
average male LC ^b^			104.0	224.4	41%	43%		
average mutated LC ^c^			46.3	209.2	44%	47%		
average wildtype LC ^d^			134.4	363.1	36%	33%		
average < 8 methylation LC ^e^			76.3	192.6	44%	44%		
average > 8 methylation LC ^f^			81.1	311.5	40%	41%		

^a^ included are cancerous female cell lines (H1975, H1993, HCC827, H2347) (*n* = 4). ^b^ included are cancerous male cell lines (H4006, H647, H1437, H2087) (*n* = 4). ^c^ included are EGFR/KRAS mutated cancerous cell lines (H1975, HCC827, H2347, H4006, H647) (*n* = 5). ^d^ included are EGFR/KRAS wildtype cancerous cell lines (H1993, H1437, H2087) (*n* = 3). ^e^ included are cancerous cell lines with methylation scores < 8 (H4006, H647, H1437) (*n* = 3). ^f^ included are cancerous cell lines with methylation scores > 8 (H1975, H1993, HCC827, H2347, H2087) (*n* = 5).

**Table 5:** Assay validation parameters Limit of Blank (LoB), Limit of Quantification (LoQ), and Limit of Detection (LoD).

Gene	LoB	LoQ	LoD
*CDH1*	0.55 RFU	2.89 RFU	69.0 pg/µL
*CDKN2Ap16*	0.50 RFU	2.78 RFU	11 pg/µL
*RASSF1A*	0.81 RFU	3.38 RFU	54.75 pg/µL
*TERT*	1.00 RFU	3.96 RFU	0.94 pg/µL
*WT1*	0.56 RFU	2.57 RFU	1.45 pg/µL

**Table 6:** Primer Sets 5′-3′ and the amplicon length of the sequencing assays; B—biotin, R = A or G (indicates a CGs in reverse assays); Y = C or T (indicates CGs).

Gene	Forward Primer	Reverse Primer	Sequencing Primer	Sequence to Be Analyzed	Amplicon Length
*CDH1*	B_GATTTTAGTAATTTTAGGTTAGAGGGTTAT	ACTAACTTCCCCAAACTCACAAATACTT	TCCCCAAACTCACAAATACTTTAC	AATTCCTACTCCACTAAAAAAAAATAC**R**TTT	236 bp
*CDKN2Ap16*	AGAGGATTTGAGGGATAGGG	B_TACCTACTCTCCCCCTCT	GGTTGGTTGGTTATTAGA	GGGTGGGG**Y**GGAT**Y**G**Y**GTG**Y**GTT**Y**GG**Y**GGTTG**Y**GGAGAGG	135 bp
*RASSF1A*	AGGGAAGGAAGGGTAAGG	B_ACTCCCCCAACTCAATAAACTCAAACTC	GGGGTTAGTTTTGTGG	TTT**Y**GTT**Y**GGTT**Y**G**Y**GTTTGTTAG**Y**GTTTAAAGTTAG	265 bp
*TERT*	GGTTAGGTAGGGTTTTTAGTGGA	B_ATACCCCAATCCCCAATCCCTC	GGTAGGGTTTTTAGTGGAT	T**Y**G**Y**GGGTATAGA**Y**GTTTAGGAT**Y**G**Y**GTTTTTTA**Y**GTGG	87 bp
*WT1*	TGGGGTAAGGAGTTTAAGGT	B_AACTCCCTACTACTCTAACTACTATA	GAGTAGGGAAGGTAGTTTAG	G**Y**GTT**Y**GGGTTT**Y**GT**Y**GTTTTTT**Y**GT**Y**G**Y**GATT	285 bp

**Table 7:** Optimized PCR parameters.

Gene	Annealing Temperature	PCR Cycles	MasterMix	Additives
*CDH1*	59 °C	35	HotStarTaq Plus MM *	1 µL CoralLoad *
*CDKN2Ap16*	59 °C	35	HotStarTaq Plus MM *	1 µL CoralLoad *
*RASSF1A*	57 °C	37	HotStarTaq Plus MM *	1 µL CoralLoad *
*TERT*	57 °C	37	HotStarTaq Plus MM *	1 µL CoralLoad *
*WT1*	57 °C	37	HotStarTaq Plus MM *	1 µL CoralLoad *

* Product of Qiagen, Hilden, Germany.

## Abbreviations

CDH1	Cadherin 1
CDKN2Ap16	Cyclin-Dependent Kinase Inhibitor 2A/p16
CGI	CpG-island
CLSI	Clinical and Laboratory Standards Institute
DMGs	differentially methylated genes
DNMTI	methyltransferase inhibitors
EGFR	epidermal growth factor receptor
EMT	Epithelial–mesenchymal transition
FFPE	formalin-fixed, paraffin-embedded
KRAS	KRAS proto-oncogene, GTPase
LC	Lung cancer
LINE-1	global interspersed nuclear elements-1
LoB	Limit of Blank
LoD	Limit of Detection
LoQ	Limit of Quantification
NSCLC	Non-small-cell lung cancer
RASSF1A	RAS-association domain family 1 isoform A
RFU	relative fluorescence units
SCLC	Small cell lung cancer
TERT	Telomerase Reverse Transcriptase
TKIs	tyrosine kinase inhibitors
WT1	Wilms’ tumor 1
